# Trajectories of children and adolescents attending a psychiatric emergency unit during the COVID-19 confinements: 2020–2022 longitudinal study

**DOI:** 10.1186/s13034-023-00619-6

**Published:** 2023-06-08

**Authors:** Marina Adrados-Pérez, Vicent Llorca-Bofí, María Mur Laín, Carla Albert Porcar, Eugènia Nicolau-Subires, Lucía Ibarra-Pertusa, Andrea Jiménez-Mayoral, Esther Buil-Reiné, Filip Budny, Belén Resa-Pérez, Vanessa Gladys Velásquez-Acebey, Laura Arenas-Pijoan, María Irigoyen-Otiñano, Jorge López-Castroman

**Affiliations:** 1 Department of Psychiatry, Santa Maria University Hospital, Lleida, Spain; 2 Institut de Recerca Biomèdica Lleida, Biological Foundations of Mental Disorders, Lérida, Spain; 3 Department of Medicine, University of Barcelona School of Medicine, Barcelona, Spain; 4 University of Lleida, Lérida, Spain; 5 University of Montpellier, IGF, CNRS-INSERM, Montpellier, France; 6 Department of Psychiatry, Nimes University Hospital, Nimes, France; 7 CIBERSAM, Madrid, Spain

**Keywords:** Suicide, Hospitalization, Children, SARS-COV-2, Emergency room, Psychiatric symptoms, Clinical evolution, Prognosis, Mental health

## Abstract

**Introduction:**

The prevalence of psychiatric disorders has not shifted widely through the COVID pandemic, except for some specific groups such as young people or women. Our objective is to examine prospectively the evolution of children and adolescents who consulted in a psychiatric emergency service during the COVID-19 confinements.

**Method:**

We collected prospective clinical information about 296 young people under 18 who visited a tertiary hospital for psychiatric reasons during the confinement periods in Spain. Clinical diagnoses, suicide attempts, hospital admissions, and pharmacological prescriptions were extracted from electronic health records through 2020, 2021, and 2022. Features of those who maintained psychiatric care and those who did not were compared.

**Results:**

Three out of four children and adolescents who visited the psychiatric emergency department during the confinements continued psychiatric care at the end of 2022. Those who did not showed better premorbid adjustment at baseline. During follow-up, diagnoses of neurodevelopmental disorders and eating disorders, as well as the dosage of psychotropic drug prescriptions, increased. The diagnoses of major depressive disorder and eating disorder at baseline were associated with attempting suicide during follow-up. Patients with internalizing symptoms were admitted earlier than those with externalizing symptoms but no differences were found in terms of suicide attempts.

**Conclusions:**

The continuity of psychiatric care after an initial emergency visit during the confinements implied greater clinical severity, as reflected by changes in clinical diagnoses and pharmacological regimens. Emergent symptoms of depression or eating disorders after social distancing or isolation could predict subsequent suicidal behavior in young populations.

## Highlights

- A large majority (76.3%) of children and adolescents presenting a psychiatric emergency during the COVID-19 confinements continued psychiatric care for at least 18 months.
- Non-continuity of psychiatric care after the index episode was associated with less severe psychiatric diagnoses and better premorbid adjustment at baseline.
- Compared to baseline, diagnoses of neurodevelopmental disorders and eating disorders, as well as psychotropic drug prescriptions, increased at the end of the study period.
- A diagnosis of major depressive disorder or eating disorder at baseline was associated with an increased risk of attempting suicide during follow-up.

## Introduction

Children and adolescents have been more affected by the psychological and social consequences of the SARS-CoV-2 (the virus the COVID-19 respiratory illness) pandemic than by its biological effects [1]. The experience of loneliness or social isolation caused by the restriction of social interactions during the pandemic seems to be an important factor [2]. Many cross-sectional studies have analyzed the impact of the pandemic on young populations [3–5]. However, longitudinal studies exploring long-term clinical outcomes in this age group are scarce and epidemiological studies show contrasting results depending on the country. Some report unchanged conditions [6–8] while others suggest worsening mental health [9–12]. Specifically, some authors have found a slight decrease in outpatient visit rates in psychiatry and emergency departments during the early pandemic and also described a subsequent growth in mental health problems and suicide-related requests from the summer of 2020 until the winter of 2021 in psychiatric emergency units [12, 13]. A recent large-scale meta-analysis confirms that variations during the COVID pandemic are mainly circumscribed to young people and women [14].

The variability of the results could be due to factors related to the context, such as the geographical areas where the studies were carried out [15] or the timing of the evaluation [10]. A prospective study of Italian adolescents observed an increase in mean anxiety scores, future uncertainty stress, and a higher frequency of maladaptive behaviors after the pandemic [16]. Conversely, the level of stress related to social domains (school attendance, romantic relationships, peer pressure) decreased. Although most minors remained stable (46%) or improved (34%), those who worsened (15%) were more likely to present disruptive behaviors such as self-harm, binge drinking, or aggressiveness. Another study in Japan reveals that in the year following the pandemic, the general suicide rate decreased first by 14% but then increased by 49% among children and adolescents [17]. Another Israeli study with comprehensive data from a large nationwide care provider reported that increasing trends of suicide attempts were temporarily interrupted by the pandemic but could be expected to continue increasing [18].

More research is lacking, but some longitudinal studies in adolescents warn that there was a higher level of mental health service use in 2021 compared to 2019: a 20% increase in new outpatients, a 39% increase in emergency visits, and a 17% increase in hospital admissions [19]. A recent systematic review supports existing concerns about the impact of the pandemic on children's mental health by noting a deterioration across the broader measures of mental health, such as an increase in global severity scores, externalizing problems and internalizing symptoms [20].

We have previously analyzed in a cross-sectional study the impact of the pandemic on urgent psychiatric care during the two COVID confinement periods in the province of Lerida (Spain) [21]. Child and adolescent visits increased by 83.5% in the second confinement compared to the first, and patients were younger, with fewer psychiatric records, and more often living with relatives than in supervised centers. Independent risk factors for suicidal behavior were being female, living with relatives, and having a diagnosis of depression. Building on these findings and the need to evaluate how the lockdowns affected children and adolescents [22], we set out to study the clinical evolution of that sample after the index episode for urgent psychiatric care.

## Method

### Sample and study design

This study was carried out at the Santa Maria University Hospital in Lleida, Spain. This hospital is the only one providing urgent psychiatric care in the province of Lleida, with a catchment area of 431,183 people [23]. The data for this study have been obtained through a longitudinal review of electronic health records (EHR) regarding all patients under 18 years of age who were assessed in the emergency room (ER) for acute psychiatric requests during the two COVID confinements in Spain (first state of emergency from March 15, 2020, to June 20, 2020; second state of emergency from October 25, 2020, to May 9, 2021 [24]). During this time, a state of alarm was decreed, schools were closed, and citizens were ordered to stay at home except for justified reasons, such as unavoidable work, purchase of food, or attendance at health centers for urgent reasons [25]. The first contact with the psychiatric emergency department during the two COVID confinements was defined as the index episode (i.e., the first assessment in the psychiatric emergency room during one of the two states of emergency). To evaluate their clinical evolution information from EHR was extracted for the years 2020, 2021, and 2022.

The authors state that all the procedures contributing to this work comply with the ethical standards of the relevant national and institutional committees on human experimentation and with the 1975 Declaration of Helsinki, revised in 2008 [26]. This study was approved by the ethics and clinical research committee of Arnau de Vilanova University Hospital (CEIC-2404).

### Clinical assessment

The following sociodemographic variables were collected at the index episode: age (years), sex (male/female), living situation (with relatives/institution), exposure to stressful life events referred to in the interview (present: ≥ 1 stressful life events within the last 12-months according to the list of events proposed by Brugha et al. [27]) and poor academic performance the previous academic year (present: ≥ 1 failed examination using self-reported grades in the latest school quarterly exams). Two experienced psychiatrists extensively reviewed the EHR of the sample for the years 2020, 2021, and 2022. The following variables were collected: psychiatric diagnoses, personality clusters, predominant externalizing/internalizing symptoms, suicidal attempts, psychiatric and general medicine emergency visits, visits to the pediatrician, and admissions in the psychiatric child and adolescent hospitalization unit.

Main psychiatric diagnoses and personality clusters, coded according to the Diagnostic and Statistical Manual of Mental Disorders, fourth edition (DSM-IV) criteria [28], were extracted from EHR. The registry of diagnoses in EHRs is used for billing and classification purposes and completed by DSM-IV-trained senior psychiatrists. Recorded psychopathological explorations at the index episode and at the end of follow-up were extensively reviewed and predominant symptoms were classified using the externalizing/internalizing framework proposed by Achenbach [29]. We used the Silverman et al. definition to code suicide attempts during follow-up: a self-inflicted, potentially harmful behavior with a non-fatal outcome for which there is evidence (either explicit or implicit) of intent to die [30].

### Pharmacological treatment

The pharmacological treatment was collected at baseline and during follow-up from the National Health System Electronic Prescription (NHSEP). Legally authorized professionals use the NHSEP as a digital health service to prescribe treatments. This system stocks the flow of information about care interventions. By using NHSEP data we could identify any patient receiving psychopharmacological treatment in public or private facilities. Antidepressants (AD) were classified in 4 groups based in the mechanism of action [31]: SSRI (sertraline, fluvoxamine, fluoxetine, paroxetine, citalopram and escitalopram), SRNA (venlafaxine, desvenlafaxine and duloxetine), tricyclics (amitriptyline, clomipramine, imipramine and nortriptyline) and atypical AD (bupropion, mirtazapine, agomelatine, vortioxetine, trazodone and agomelatine). Antipsychotics (AP) were classified in 4 groups according to the data-driven taxonomy proposed by McCutcheon et al. [32]: muscarinic (chlorpromazine, clozapine, olanzapine, quetiapine, clotiapine), adrenergic/Low DA (aripiprazole, asenapine, cariprazine, lurasidone), serotonergic/dopaminergic (haloperidol, risperidone, paliperidone) and dopaminergic (amisulpride, sulpiride, pimozide). Dose equivalent criteria were used to estimate daily drug intake for AD (fluoxetine equivalents) [33], AP (chlorpromazine equivalents) [34], and benzodiazepines (diazepam equivalents) [35]. Categorical data (yes/no) was collected for other psychopharmacological groups: anticonvulsants (valproate, carbamazepine, lamotrigine, topiramate, gabapentin or pregabalin), noradrenergic stimulants (methylphenidate or amphetamines), noradrenergic non-stimulants (atomoxetine), alpha-2 adrenergic antagonists (clonidine or guanfacine), anticholinergics (biperiden) and lithium.

### Follow-up care

Follow-up care after the index visit to the emergency department was considered a proxy of clinical severity. By doing so, we assume that patients that did not have any psychiatric contact in the aftermath of the emergency visit present less severe disorders. Follow-up care was endorsed for participants fulfilling at least one of the following criteria during the study: (i) at least 3 scheduled psychiatric or pediatrician consultations; (ii) any visit to the psychiatric emergency room; (iii) any admission to the child and adolescent psychiatry hospitalization unit; (iv) any active psychopharmacological prescription. End of care implied fewer than 3 ambulatory visits, no psychiatric emergency room visit, and no hospitalizations or psychopharmacological treatment [36]. The term “end of care”, in this case, reflects the absence of mental health care after the index episode and may imply the absence of mental health needs.

### Statistical analyses

Statistical analyses were performed using the IBM-SPSS v.23 statistical package. Continuous data are expressed as mean ± standard deviation while categorical data are presented as percentages. Normal distribution was evaluated using the Shapiro–Wilk test. Differences in demographic and clinical characteristics between patients that had follow-up psychiatric care and those who did not were assessed using Chi-square tests on categorical data and *t*-test (or non-parametric Mann–Whitney *U*) on continuous data. Comparative analyses were performed between the index episode and paired data at the end of the follow-up. Statistical analyses for intra-subject comparisons were performed using Wilcoxon signed-rank test for continuous data and the McNemar test for categorical data. Kaplan–Meier survival curves and Cox regression analysis were used to estimate the time to suicidal attempt/hospitalization and compare the median time to relapse between patients with internalizing and externalizing symptoms at the index episode. The following covariables were used for the Cox regression analysis: age, gender, psychiatric diagnosis, and psychopharmacological treatment at index episode. Univariate analyses were performed to explore whether sociodemographic and clinical variables at the index episode were associated with later suicide attempts. Fisher’s exact test (FET) provided the significance and the odds ratios (OR) and their 95% confidence intervals (CI) provided the effect size. The significant variables (p < 0.05) in the univariate analyses were included in a multivariate logistic regression model. Type I error was set at the usual value of 5% (alpha = 0.05) with a two-sided approximation.

## Results

### Demographic and clinical characteristics at index episode

A total of 296 patients were included. The mean age was 15.3 years (± 1.7). Two-thirds (66.6%) of the participants were females (Table 1). The majority of the patients visited the ER during the second lockdown (80.7%) and were living with relatives (89.4%). 69,9% had records of psychiatric follow-up and 56.7% had records of psychopharmacological treatment at the index episode. The main psychiatric diagnosis at the index episode were depressive disorders (19.5%) and impulse control disorders (19.2%). Many patients (43.3%) had dysfunctional personality traits or a diagnosis of personality disorder. 41% presented externalizing symptoms and 24.3% contacted the emergency department due to a suicide attempt.

**Table 1:** Baseline demographic and clinical variables of the sample and comparison between contact and non-contact patients

	Total sample (N = 296)	Non-contact patients (n = 70)	Contact patients (n = 226)	p-value
Age at index episode, mean (SD)	15.3 (1.7)	15.2 (1.6)	15.4 (1.7)	0.485
Females, n (%)	196 (66.6)	51 (74.3)	145 (64.2)	0.133
Visit during the COVID-19 period, n (%)				
First lockdown	57 (19.3)	7 (10.0)	50 (22.1)	0.027*
Second lockdown	139 (80.7)	62 (90.0)	176 (77.9)	
Living, n (%)				
With relatives	238 (89.4)	57 (81.4)	181 (80.1)	0.953
Institution	58 (19.8)	13 (18.5)	45 (19.9)	
Stressful life events, n (%)	79 (26.6)	15 (21.4)	64 (28.4)	0.128
Poor school performance, n (%)	147 (49.6)	19 (27.1)	128 (56.9)	** < 0.001**
Previous psychiatric follow-up, n (%)	207 (69.9)	26 (37.1)	181 (80.1)	< 0.001*
Previous psychopharmacological treatment at index episode, n (%)	168 (56.7)	15 (21.4)	153 (68.0)	< 0.001*
Psychiatric diagnoses, n (%)				
Neurodevelopmental disorders	28 (9.4)	1 (1.4)	27 (11.9)	< 0.001*
Intellectual disabilities	10 (3.3)	0 (0)	10 (4.4)	0.124
Psychotic disorders	8 (2.7)	0 (0)	8 (3.5)	0.205
Bipolar disorders	3 (1.0)	0 (0)	3 (1.3)	0.977
Depressive disorders	58 (19.5)	5 (7.14)	53 (23.4)	0.002*
Anxiety disorders	20 (6.7)	6 (8.7)	14 (6.2)	0.610
Adjustment disorders	30 (10.1)	14 (20.0)	16 (7.1)	< 0.001*
Eating disorders	32 (10.8)	8 (11.42)	24 (10.6)	0.976
Impulse control deficit	57 (19.2)	13 (18.3)	44 (19.4)	0.867
Substance use disorder	29 (9.7)	7 (10.0)	22 (9.7)	0.947
None	21 (7.1)	16 (22.8)	5 (2.2)	< 0.001*
Internalizing and externalizing framework				
Internalizing	173 (58.4)	33 (47.2)	140 (61.9)	0.028*
Externalizing	123 (41.5)	37 (52.8)	86 (38.1)	
Personality clusters, n (%)				
None	168 (56.7)	42 (60.0)	123 (54.4)	0.570
Cluster A	11 (3.7)	1 (1.42)	10 (4.4)	0.251
Cluster B	82 (27.7)	14 (20.3)	68 (30.1)	0.134
Cluster C	28 (9.4)	3 (4.2)	25 (11.1)	0.144
Suicidal attempt, n (%)	72 (24.3)	5 (7.1)	67 (29.3)	** < 0.001**

A majority (n = 226, 76.3%) of the 296 patients that received acute psychiatric care during the confinements had follow-up care at the end of the follow-up (Table 1). Patients who ended psychiatric care were more likely to have visited the ER during the second confinement (90.0% vs 77.9%; p = 0.027), had fewer problems with academic performance (27.1% vs 56.9%; p < 0.001), as well as fewer psychiatric records (37.1% vs 80.1%; p < 0.001) and psychopharmacological prescriptions (21.4% vs 68.0%; p < 0.001). They were also more likely to be diagnosed with no psychiatric disorder (22.8% vs 2.2%; p < 0.001) or adjustment disorder (20.0% vs 7.1%; p < 0.001), and less likely to be diagnosed with a neurodevelopmental disorder (1.4% vs 11.9%; p = 0.002) or major depressive disorder (7.14% vs 23.4%; p = 0.002). In addition, the end of care was associated with more externalizing symptoms (52.8% vs 38.1%; p = 0.028) and fewer suicide attempts (7.1% vs 29.3%; p < 0.001) at the index episode.

### Follow-up care

In total, 226 patients had follow-up psychiatric care after the index visit. Psychiatric care lasted a minimum of 18 months and a maximum of 33 months with a mean duration of 27.8 ± 4.3 months. Patients under follow-up care were aged on average 15.43 years (± 1.77) and were more often females (64.2%). Table 2 describes the clinical and pharmacological trajectories in this subgroup.

**Table 2:** Clinical features and pharmacological treatment at the index episode and at the end of the follow-up of contact patients (n = 226)

	Index episode n (%)	End of follow-up n (%)	p-value
Clinical features			
Psychiatric diagnoses			
Neurodevelopmental disorders	27 (11.9)	49 (21.6)	0.001*
Intellectual disabilities	10 (4.4)	7 (3.09)	0.620
Psychotic disorders	8 (3.5)	8 (3.5)	1.000
Bipolar disorders	3 (1.3)	2 (0.9)	0.652
Depressive disorders	53 (23.4)	55 (24.3)	0.825
Anxiety disorders	14 (6.2)	10 (4.4)	0.160
Adjustment disorders	16 (7.1)	7 (3.1)	0.086
Eating disorders	24 (10.6)	44 (19.4)	0.012*
Impulse control deficit	44 (19.4)	13 (5.7)	< 0.001*
Substance use disorder	22 (9.7)	12 (5.3)	0.074
None	5 (2.2)	19 (8.4)	0.002*
Internalizing and externalizing framework			
Internalizing	140 (61.9)	125 (60.4)	0.152
Externalizing	86 (38.1)	82 (39.6)	
Personality clusters			
None	123 (54.4)	161 (71.2)	< 0.001*
Cluster A	10 (4.4)	9 (3.9)	0.814
Cluster B	68 (30.1)	53 (23.5)	0.088
Cluster C	25 (11.1)	3 (1.3)	< 0.001*
Pharmacological Treatment			
Any psychopharmacological drug	155 (68.6)	177 (78.3)	0.014*
Any antidepressant	95 (42.0)	122 (54.0)	0.002*
AD equivalent dose, mg/d fluoxetine (SD)	30.6 ± 15.4	41.9 ± 24.7	< 0.001*
Switching AD family	40/95 (41.6)		–
AD monotherapy to polytherapy	9/95 (9.4)		–
Any antipsychotic	119 (52.7)	141 (62.4)	0.012*
LAI	9 (4.0)	17 (7.5)	0.057
AP equivalent dose, mg/d chlorpromazine (SD)	192.4 ± 170.1	232.5 ± 219.9	0.112
Switching AP family	49/119 (41.1)		–
AP monotherapy to polytherapy	21/119 (17.6)		–
Any benzodiazepine	68 (30.1)	60 (26.9)	0.445
BZD equivalent dose, mg/d diazepam (SD)	21.4 ± 15.9	24.5 ± 19.3	0.241
Other drugs			
Anticonvulsants	6 (2.7)	43 (19.0)	< 0.001*
Noradrenergic stimulants	5 (2.2)	9 (4.0)	0.219
Noradrenergic nonstimulants	2 (0.8)	3 (1.3)	0.417
Alpha-2 adrenergic antagonists	3 (1.3)	13 (5.8)	0.002*
Anticholinergics	1 (0.4)	4 (1.8)	0.250
Lithium	0 (0)	2 (0.9)	0.890

Throughout the follow-up, the number of patients without psychiatric diagnoses (2.2% vs 8.4%; p = 0.001) and without personality disorders increased (54.4% vs 71.2%; p < 0.001). There was a significant increase in neurodevelopmental disorders (11.9% vs 21.6; p = 0.001) and eating disorders (10.6% vs 19.4%; p = 0.012) along with a decrease in impulse control disorders (19.4% vs 5.7%; p < 0.001). In addition, we found a decrease in cluster C personality disorders (11.1% vs 1.3%; p < 0.001).

The proportion of pharmacological prescriptions at the end of the study was higher than at the index episode (68.6% vs 78.3%; p = 0.014). Specifically, we found an increase in the proportion (42.0% vs 54.0%; p = 0.002) and dose (30.6 ± 15.4 vs 41.9 ± 24.7 mg/d of fluoxetine; p < 0.001) of prescribed AD together with an increase in the proportion of AP (52.7% vs 62.4%; p = 0.012), anticonvulsants (2.7% vs 19.0%; p < 0.001) and alpha-2 adrenergic antagonists (1.3% vs 5.8%: p = 0.002) prescribed.

Looking at medication variations during follow up, we observed that 41.6% switched the AD (37.5% SSRI to SNRI, 32.5% SSRI to SSRI, 15% SSRI to tricyclic, 12.5% SSRI to atypical AD and 2.5% SNRI to SNRI) and 9.4% changed from AD monotherapy to AD polytherapy. 41.1% changed the AP (26.5% serotonergic/dopaminergic to muscarinic, 22.4% same family of AP, 12.2% Serotonergic/Dopaminergic to Adrenergic/Low DA, 12.2% Muscarinic to Adrenergic/Low DA, 6.1% other changes) and 17.6% changed from AP monotherapy to AP combination therapy.

### Suicide attempts at baseline and during follow-up

72 patients (24.3%) made a suicide attempt at the index episode and 87 patients (29.3%) attempted suicide during follow-up. Regarding their index visit, patients who attempted suicide during follow-up were more likely to have visited the ER during the first lockdown (27.6% vs 15.8%; p = 0.016) and be diagnosed with major depressive disorder (43.6% vs 22.9%; p =  < 0.001) or eating disorder (17.2% vs 6.6%; p = 0.009) than those who did not. Individuals having attempted suicide at the index episode were not more likely to repeat the attempt during the follow-up (p = 0.150). Both diagnoses, but not the lockdown period of the index episode, emerge as independent risk factors for suicide attempts in the multivariate logistic regression: depressive disorders (OR: 3.109 [1.178 to 6.001]) and eating disorders (OR: 1.557 [1.059 to 2.813]) (Table 3). To better understand the potential of mediator factors in the associations between depression and eating disorders with subsequent suicide, previous psychiatric follow-up (yes/no) and social restrictions according to the period of the ER visit (fist/second lockdown) were analyzed. Following Baron & Kenny’s steps for mediation analysis [37], neither previous psychiatric follow-up nor period of the visit served as mediating factors between depression or eating disorders and suicidal attempts.

**Table 3:** Bivariate and multivariate logistic regression on suicidal behavior during follow-up

	Non suicidal behavior (n = 209; 70.6%) n (%)	Suicidal behavior (n = 87; 29.4%) n (%)	p-value	OR univariate	CI
Age at index episode, mean (SD)	15.37 (1.78)	15.44 (1.63)	0.779		
Females, n(%)	135 (65.6)	60 (69.0)	0.592		
Visit during the COVID-19 period					
First lockdown	33 (15.8)	24 (27.6)	0.016*	2.003	− 0.890 to 3.718
Second lockdown	176 (84.2)	63 (72.4)			
Living					
With relatives	169 (80.9)	69 (79.3)	0.754		
Institution	39 (19.2)	18 (20.7)			
Stressful life events	40 (27.0)	28 (32.2)	0.457		
Poor school performance	86 (58.1)	46 (52.9)	0.496		
Previous psychiatric follow-up	177 (84.7)	70 (80.5)	0.393		
Previous psychopharmacological treatment at index episode	94 (64.4)	64 (73.6)	0.192		
Psychiatric diagnoses at index episode					
Neurodevelopmental disorders	44 (22.0)	10 (11.5)	0.075		
Intellectual disabilities	6 (2.0)	0 (0)	0.624		
Psychotic disorders	7 (3.3)	1 (1.1)	0.502		
Bipolar disorders	2 (1.0)	0 (0)	0.706		
Depressive disorders	48 (22.9)	38 (43.6)	< 0.001*	4.513	1.801 to 10.764
Anxiety disorders	20 (9.5)	4 (4.5)	0.232		
Adjustment disorders	18 (8.6)	4 (4.5)	0.338		
Eating disorders	14 (6.6)	15 (17.2)	0.009*	2.782	1.057 to 7.323
Impulse control deficit	18 (8.6)	5 (5.7)	0.293		
Substance use disorder	13 (6.2)	3 (3.4)	0.412		
None	18 (8.6)	7 (8.0)	0.944		
Internalizing and externalizing framework at index episode					
Internalizing	130 (62.2)	55 (63.2)	0.896		
Externalizing	79 (37.8)	32 (36.8)			
Personality clusters at index episode					
None	161 (77.0)	63 (72.4)	0.640		
Cluster A	1 (0.5)	1 (1.1)	0.623		
Cluster B	44 (21.1)	23 (26.4)	0.402		
Cluster C	3 (1.4)	0 (0)	0.287		
Suicidal behavior at index episode	46 (22.0)	26 (29.8)	0.150		

Multivariate logistic regression model				
Variable	Wald *X*^2a^	p-value	OR corrected	CI
First step				
Depressive disorders	13.215	<0.001*	3.109	1.178 to 6.001
Eating disorder	9.688	0.003*	1.557	1.059 to 2.813

^a^The Hosmer–Lemeshow test was not significant (X^2^ = 13.094, df = 5, p = 0.614)

^*^ p < 0.05

**Fig. 1:**
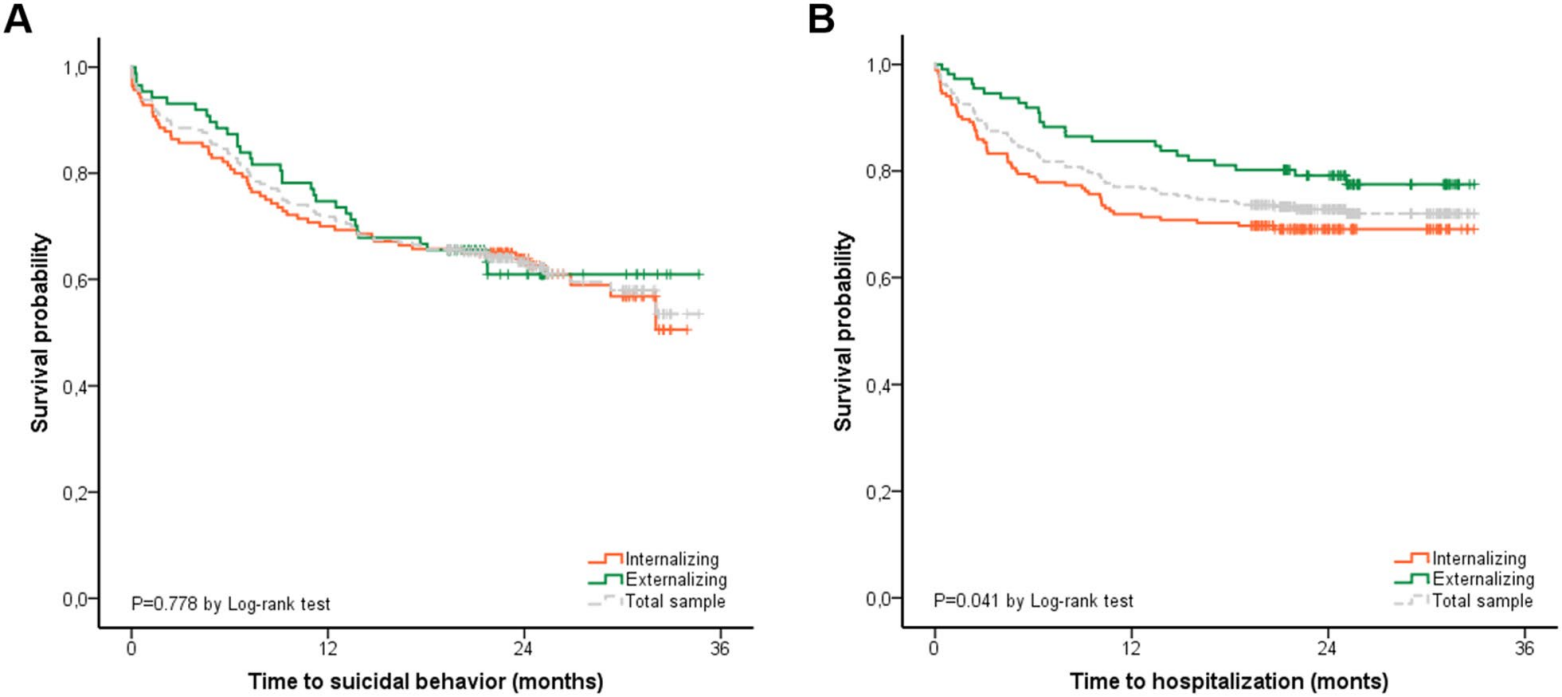
Kaplan–Meier curves and Long-rank test for suicidal behavior (**A**) and hospitalization (**B**) comparing internalizing vs externalizing symptoms at index episodes

In the survival analysis, the rate of patients making incident attempts was 28.4% one year after the index episode and 36.8% throughout the duration of the study (Fig. 1A). No differences were found in suicide attempts between internalizing and externalizing patients (p = 0.778) (Fig. 1A).

### Comparison between hospitalized and non-hospitalized patients

112 patients (37.8%) were admitted to the hospital at the index episode and 81 patients (27.4%) were admitted during the follow-up. Patients with internalizing symptoms were more likely to be hospitalized than those with externalizing symptoms (30.9% vs 17.1%; p = 0.009). In the survival analysis, internalizing patients were admitted earlier after the index visit (p = 0.041) (Fig. 1B). The Cox regression analysis adjusted by covariables replicated that internalizing symptoms at baseline were significantly associated with hospitalization (HR = 1.93, p = 0.001).

Patients admitted to the hospital during follow-up were more likely to have psychiatric records (79.1% vs 51.4%; p < 0.001), psychopharmacologic treatment (76.3% vs 49.8%; p < 0.001), internalizing symptoms (65.8% vs 47.3%; p = 0.005), and suicide attempts at index episode (30.2% vs 19.8%; p = 0.048) compared to non-hospitalized patients. They also had fewer diagnoses of impulse control disorders (4.3% vs 13.5%; p = 0.023). In the multivariate logistic regression, only the existence of psychiatric records was independently associated with hospitalization (OR: 2.760 [1.102 to 4.031]).

## Discussion

This study evaluates the clinical evolution over more than 18 months of a sample of minors that received acute psychiatric care during the confinement periods in Spain. Three out of four had follow-up psychiatric care and, among them, depressive disorders, eating disorders, increased the risk of suicide attempts throughout the follow-up. Indeed, a clinical worsening was observed during follow-up, with an increase of neurodevelopmental and eating disorders and higher dosages of pharmacological treatments.

The end of psychiatric care after the emergency visit implied better premorbid adaptation in terms of mental health and school level, more externalizing symptoms, adaptative related disorders and no lower suicidal behavior. The index visit requiring acute care was probably related to the social distancing imposed against the SARS-COV-2 virus [22, 38, 39]. This aspect is consistent with the literature that indicates fluctuations associated with the tightening of confinement measures and differential effects among young people [40].

The 226 patients who had follow-up psychiatric care after the emergency visit presented greater clinical severity in terms of suicidal behavior and clinical profile at the index episode. This finding suggests that despite the epidemiological situation, it has been possible to maintain contact with the most seriously ill patients [41]. The diagnoses of neurodevelopmental disorders and eating disorders increased among them until the end of 2022. It should be noted that studies of children and adolescents during the pandemic show that those with neurodevelopmental disorders or special educational needs had the highest levels of poor mental health and the majority of autistic children experienced a worsening of their pre-pandemic psychiatric diagnoses and/or the development of new psychiatric symptoms [42]. Other researchers have already pointed out the greater vulnerability of children with neurodevelopmental disorders since the pandemic led to increased social distance and even the interruption of support measures that are essential for the good management of these disorders [43]. On the other hand, preliminary studies indicate an increase in the prevalence of eating disorders after the pandemic [44, 45], often requiring hospital admissions [46]. Unlike other studies [47], we did not find a significant increase in substance use disorders. Importantly, rather than the conditions of confinement or lockdown, the risk of suicidal behavior during follow-up seems to be carried by the development of specific mental health problems, in particular, eating disorders and MDD.

In our study, predominant externalizing or internalizing symptoms at index episode were not associated with suicidal attempts during the follow-up. However, recidivism was associated with depression, a diagnosis that is strongly linked to suicidal behavior [48]. According to our data, a diagnosis of depression or eating disorder in the ER during the confinements increased the risk of a subsequent suicide attempt by 3.1 and 1.5 respectively. Another study by our group has already shown an increase in suicidal behavior in patients of all ages with eating disorders during the period of confinement due to the pandemic [49] and other groups have also verified this [50]. These findings are highly relevant as consistent longitudinal studies on suicide attempts and recidivism in youth during the pandemic are lacking. Furthermore, specific strategies in these population have been associated with improvements in suicide mortality [51].

When comparing the periods before and during the pandemics, a recent systematic review points out a non-significant upward trend in suicidal behaviors among the general population and in the emergency setting [5]. Other studies have evaluated recidivism in adults [52–54], but studies in minors are scarcer. Importantly, recidivism in young patients within 12 months of a suicide attempt has been associated in the literature with substance abuse, nonaffective psychotic disorders, chronic medical conditions, or a history of sexual abuse [55]. In Catalonia, the Suicide Risk Prevention Code identifies and offers preferential visits to all users presenting suicidal behavior [56]. The 2019 results of this program list 465 episodes of suicidal behavior between the ages of 12 and 17, with 15.7% reattempts (and 2.7% of them leading to death). Recurrence of self-harm is common, with 15–25% of adolescents treated in a hospital for an episode of self-harm returning for treatment within 12 months [57].

Another severity criterion was the need for hospitalization during the observed period. 25.3% of patients with externalizing pathology were admitted in the following year and 39.1% at the end of follow-up. In contrast, 30% of patients with internalizing symptoms were admitted in the following year and 36.1% at the end of the follow-up, but there was a shorter time interval from emergency care to admission. This point is relevant considering that hospital admission was partially restricted during the pandemic as a response to the health crisis. A French study reports a decrease in admissions until the end of 2020 for suicidal behavior [58]. Hospital admission can be considered a criterion of severity, especially in suicide attempts [48]. Studies on hospital admissions of minors for suicidal behavior during the pandemic period are scarce. A study on Spanish adolescents admitted to the child and adolescent unit for suicidal behavior between February 2021 and June 2022 showed that older age, and particularly the presence of non-suicidal self-harm, was closely related to a diagnosis of personality disorder [59]. Another Italian study compared < 18 years inpatients between March 2020 and June 2021 with those admitted in 2018–2019, evidencing a slight increase of admissions during the pandemics. Patients admitted during the pandemic were more likely to have a family history of psychiatric disorders and a personal history of physical disorders, to present suicidal risk and to be diagnosed with an externalizing disorder [60].

## Limitations and strengths

These results must be interpreted with some limitations in mind. First, the data presented here come from EHR and relies on clinical diagnoses made by different psychiatrists. However, this is a single-center study and all psychiatrists working in the emergency department have been trained with similar diagnostic criteria based on DSM-IV. Second, symptom severity was not measured, and some relevant variables, such as gender identity, were not collected. Suicide attempts and hospital admissions were used as a proxy of clinical severity. Although these naturalistic measures can be compared with other centers, they might be conditioned by the availability of local resources, especially during the pandemic. Furthermore, the definition of suicide attempt relies on the presence of intent to die, which can be difficult to assess. Non-suicidal self-harm, which we could not evaluate in our sample, also carries a certain risk for suicidal behavior [61]. Finally, this sample is representative of a population seeking acute care services and results may not be generalizable to other settings.

Despite being a relatively small sample, it represents the entire population under 18 years of age who visited the only psychiatric emergency department in the province. Furthermore, data regarding pharmacological prescriptions is exhaustive. Second, the clinical data of patients receiving follow-up care extends longitudinally for a long period (18 to 33 months) after the pandemic period and allows a detailed characterization of the sample.

## Conclusions

In this longitudinal study, a large majority of children and adolescents presenting a psychiatric emergency during the COVID-19 confinements had follow-up psychiatric care for at least 18 months. These patients showed worse premorbid adjustment and higher internalizing symptoms. During the follow-up, depressive and eating disorders at index episode increased the risk of suicidal attempt, and presenting internalizing symptoms was associated with earlier hospital admissions.

## Data Availability

The databases and the clinical history are digitized.
